# Lack of Increased Immediate Early Gene Expression in Rats Reinstating Cocaine-Seeking Behavior to Discrete Sensory Cues

**DOI:** 10.1371/journal.pone.0072883

**Published:** 2013-09-17

**Authors:** Matthew D. Riedy, Kristen A. Keefe

**Affiliations:** Department of Pharmacology and Toxicology and Program in Neuroscience, The University of Utah, Salt Lake City, Utah, United States of America; Utrecht University, The Netherlands

## Abstract

Drug-seeking behavior elicited by drug-associated cues contributes to relapse in addiction; however, whether relapse elicited by drug-associated conditioned reinforcers (CR) versus discriminative stimuli (DS) involves distinct or overlapping neuronal populations is unknown. To address this question, we developed a novel cocaine self-administration and cue-induced reinstatement paradigm that exposed the same rats to distinct cocaine-associated CR and DS. Rats were trained to self-administer cocaine in separate sessions. In one, a DS signaled cocaine availability; in the other, cocaine delivery was paired with a different CR. After extinction training and reinstatement testing, where both cues were presented in separate sessions, rats were sacrificed and processed for cellular analysis of temporal activity by fluorescent *in situ* hybridization (CatFISH) for activity regulated cytoskeleton-associated protein (*Arc*) mRNA and for radioactive *in situ* hybridization for *Arc* and *zif268* mRNAs. CatFISH did not reveal significant changes in *Arc* mRNA expression. Similar results were obtained with radioactive *in situ* hybridization. We have shown that while rats reinstate drug seeking in response to temporally discrete presentations of distinct drug-associated cues, such reinstatement is not associated with increased transcriptional activation of *Arc* or *zif268* mRNAs, suggesting that expression of these genes may not be necessary for cue-induced reinstatement of drug-seeking behavior.

## Introduction

Drug addiction is defined as uncontrollable, compulsive drug seeking and use in the face of negative consequences (http://www.nida.nih.gov/PublishedArticles/Essence.html). Current theories posit that addiction reflects the strong association of previously neutral stimuli with drug-seeking and -taking behaviors, and the subsequent ability of these stimuli to elicit such behavior [1]–[5]. In humans, exposure to drug-associated sensory stimuli induces intense drug craving, a reliable precursor to relapse [5]–[7]. Understanding the neural mechanisms that encode drug-cue-behavior associations during initial drug use and how subsequent exposure to drug-associated stimuli influences brain activity and behavior is critical for designing successful interventions for drug addiction and relapse.

At least two types of associations are involved in stimulus-associated learning. Discriminative stimuli (DS) indicate the opportunity (DS+), or lack thereof (DS−), to obtain a reinforcer, whereas conditioned reinforcers (CR) come to serve as reinforcers themselves due to their repeated pairing with primary reinforcement. Through the course of drug use and drug-seeking behavior, people are repeatedly exposed to both types of drug-stimulus associations. There are likely similarities and differences in the neural circuits underlying drug-seeking and drug-taking behavior mediated by exposure to CRs and DS [2]. Prior studies investigating the neural substrates of CR-maintained drug-seeking behavior using lesion and pharmacological approaches have produced substantial evidence for a neural circuit involving the basolateral amygdala (BLA) [8], [9], prefrontal cortex (PFC) [8], [10], [11], nucleus accumbens core (NAc) [12], hypothalamus [13], and ventral tegmental area (VTA) [14] in mediating the ability of CRs to maintain drug-seeking behavior [15]. However, studies examining changes in immediate early gene (IEG) expression have not always consistently identified activation of these same brain regions, particularly the NA and PFC, associated with CR-mediated reinstatement of drug seeking [16]–[19]. With the exception of the BLA, then, the extent to which different brain regions are activated by CRs maintaining drug-seeking behavior is not clear.

Other studies have examined neural circuits underlying DS-induced reinstatement of drug-seeking behavior [16], [20]. However, in a number of these studies CRs are presented in close temporal proximity to the DS (e.g. on every DS-cued trial) or the DS has likely acquired CR properties by virtue of coincident exposure with primary reinforcement. Despite this caveat, DS presented in isolation can elicit reinstatement of drug-seeking behavior [21], [22]. Such studies implicate the BLA, as well as the NA shell (vs. the core) as critical nodes of the neural circuit supporting the ability of DS to reinstate drug-seeking behavior. Further delineation of the neural circuitry involved in the ability of discrete, isolated DS to elicit drug-seeking behavior is lacking. Furthermore, a direct examination of the extent to which exposure to CR vs. DS activates the same or different neuronal ensembles has not been reported. Therefore, a goal of the present work was to develop a cocaine self-administration training protocol that would develop segregated DS and CR associations in the same animal in order to identify the influence of these distinct types of associated-cues on reinstatement of drug-seeking behavior. Development of this approach, then, importantly provides a behavioral model in which to assess whether overlapping populations of neurons are involved in the formation and/or retrieval of distinct associations in the same animal.

The expression of IEGs has been used to map neural circuits involved in particular behaviors [23] including cue-induced reinstatement of drug-seeking behavior [16], [24]–[28]; however, the time course over which different IEGs are expressed and examined in studies conducted to date may obscure the extent to which it is the exposure to the drug-associated cue *per se* that is resulting in IEG activation. Also, prior studies examining IEG expression in the context of cue-induced reinstatement of drug-seeking behavior have not afforded the opportunity to examine whether discrete types of drug-associated cues activate overlapping populations of neurons. Cellular analysis of temporal activity by fluorescent *in situ* hybridization (catFISH) for activity-regulated cytoskeleton-associated protein (*Arc*) mRNA is a labeling technique that allows a tight temporal relation to be drawn between a behavioral event and resulting gene expression profile. CatFISH further allows one to determine whether the same neuronal populations within a brain region are activated by two temporally discrete behavioral events [29], [30]. Prior studies with the catFISH approach have shown that *Arc* mRNA transcription can be detected within 3–5 min of activation in forebrain regions, including hippocampus [29], [31] and striatum [31]–[33]. The *Arc* mRNA is subsequently transported into the cytoplasm and dendrites within 30 min of transcriptional activation [29], [32]. In the absence of continued neuronal activation, *Arc* mRNA transcriptional activation returns to basal levels within 60 min [29], [32]. Thus, cells with discrete foci of *Arc* mRNA in the nucleus are those activated 3–5 min prior to sacrifice of the animal; cells with *Arc* mRNA in the cytoplasm are those activated approximately 30 min prior to sacrifice; and cells with *Arc* mRNA expression in both nuclear foci and cytoplasm are activated at both time points relative to sacrifice [29], [32]. The second goal of the present work, therefore, was to detect *via* catFISH analysis of *Arc* mRNA expression neuronal populations activated during reinstatement of cocaine-seeking behavior induced by discrete drug-associated CRs and DS.

## Materials and Methods

### Ethics Statement

This study was conducted in strict accordance with the *Guide for the Care and Use of Laboratory Animals* of the National Institutes of Health. The protocol was approved by the Institutional Animal Care and Use Committee at the University of Utah (approved protocol #08–04004). All surgery was performed under ketamine/xylazine anesthesia, and all efforts were made to minimize suffering.

### Subjects

Adult male Sprague-Dawley rats (n = 36; ∼300g; Charles River Laboratories, Raleigh, NC) were food restricted (∼15 g chow/day) for two days and then trained to lever press (>300 presses/16 hrs) on a free operant schedule (FR1) for food reinforcement (45-mg pellet) in operant training chambers (Coulbourn Instruments) housed within light- and sound-attenuating boxes. Subsequently, rats were randomly assigned to treatment groups. Five cohorts were run, consisting of one animal in each of the following groups: experimental (EXP; n = 5), stimulus novelty control (NC; n = 5), stimulus control (SC; n = 5), and drug control (DC; n = 5). During training, experimental rats engaged in lever pressing behavior for intravenous cocaine (0.25 mg cocaine-HCL/50 µL 0.9% saline/2-sec infusion; dose calculated as the salt) in response to a DS and for response-contingent exposure to a complex CR in distinct segments of daily sessions. Novelty control animals were placed in chambers, but not exposed to stimuli or given infusions. Drug controls received cocaine infusions simultaneously with EXP rats, but were not exposed to sensory stimuli. Stimulus controls received exposure to the DS and CR stimuli simultaneously with EXP rats, but received saline (50 µL 0.9% saline/2-sec) infusions. An additional cohort (n = 10) consisted entirely of EXP animals. After the EXP rat had achieved self-administration stability criterion (<15 coefficient of variation in cocaine infusions across three consecutive sessions), all rats in a cohort then received identical extinction training and exposure to the CR and DS on the reinstatement test-day. Finally, age-matched, caged-control animals (CC; n = 6) were maintained for the duration of training and extinction of each cohort and were sacrificed on test day immediately prior to EXP animals.

### Surgery

Rats (EXP, SC, DC) were implanted with jugular vein catheters constructed from 22-gauge guide cannulae, silastic tubing, and monofilament mesh under ketamine/xylazine anesthesia (90/10 mg/kg, i.p.). Rats recovered for four days with free access to food and water. On the 5th day rats were food restricted as described above, and on the 6th day, cocaine self-administration training began.

### Cocaine self-administration

Training occurred during the dark phase of the light-dark cycle in chambers containing one active and one inactive lever, tricolor LEDs above each lever, and a wall-mounted house light and speaker. Each session began with a 5-min delay and lasted for four hr and 35 min. EXP rats had a two-hr DS or CR session, a 30-min time out, and the other two-hr DS or CR session. DS/CR session order was balanced across subjects.

### Discriminative stimulus

**Figure 1:**
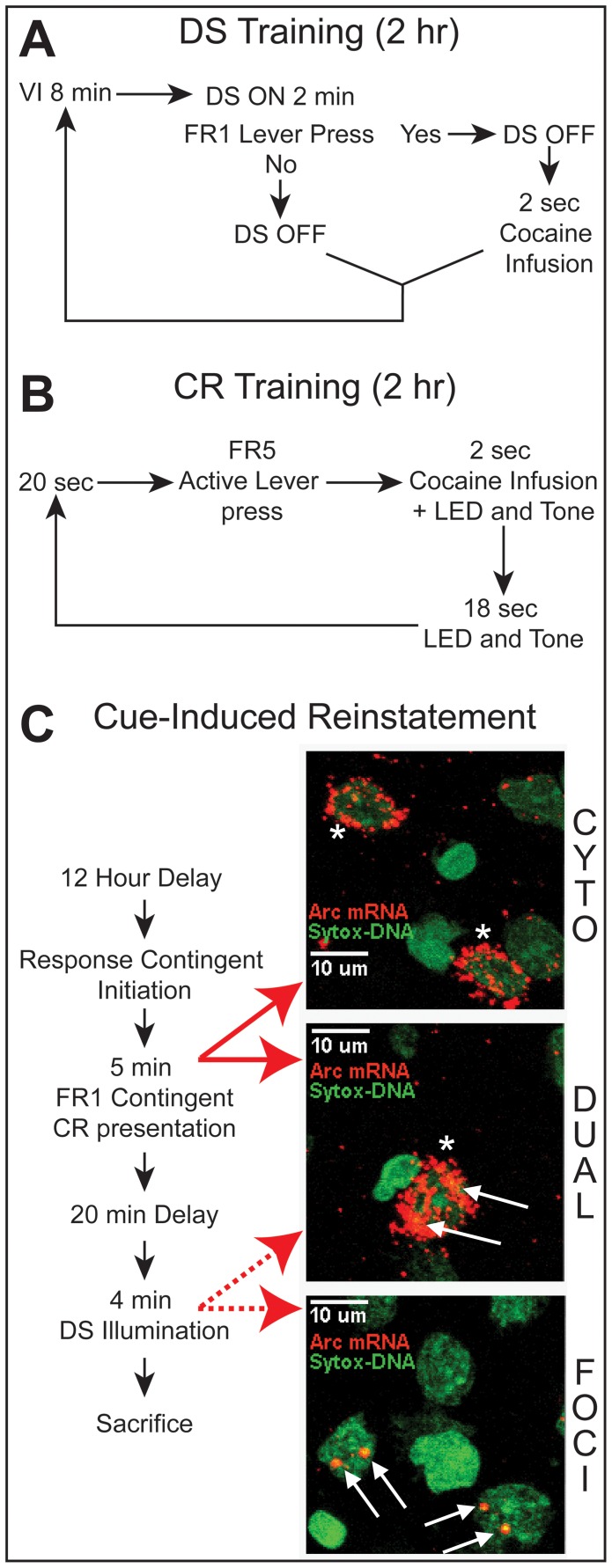
Cocaine self-administration training, cue-induced reinstatement, and catFISH expression profiles. Each session began with a 5-min delay and lasted for four hours and 35 min. EXP rats had a two-hr DS or CR session followed by a 30-min time out and the alternate session. (**A**) *Discriminative stimulus training* The DS (house light) was illuminated for up to two min. Contingent on FR1 active-lever pressing, the DS extinguished and the rat received a cocaine infusion followed by a variable-interval time out (VI; mean  = 8 min). (**B**) *Conditioned reinforcer training* EXP rats received a cocaine infusion paired with a complex CR (illumination of tricolor LEDs and tone) contingent on FR5 active-lever pressing. After cocaine infusion and CR exposure there was a 20-sec non-signaled time out. (**C**) *Cue-induced reinstatement* After extinction, rats remained in the operant chambers overnight. The next morning, contingent on an EXP active-lever press, the CR was presented for 5 sec. For the next 5 min, the CR was presented for two sec, contingent on FR1 active-lever pressing. Next, there was a 20-min time out. Then the DS was illuminated for two min, extinguished for one min, re-illuminated for two min, and finally extinguished. (**C**) *CatFISH analysis examples* Neurons showing one or two intranuclear foci of *Arc* mRNA expression (white arrows; “**FOCI**”), cytoplasmic *Arc* mRNA expression (white asterisks; “**CYTO**”), or both intranuclear and cytoplasmic *Arc* mRNA expression (“**DUAL**”). Neurons expressing only foci of *Arc* mRNA (i.e. those activated 5 min prior to sacrifice) are referred to as “DS-associated,” neurons expressing only cytoplasmic *Arc* mRNA (i.e. those activated 30 min prior to sacrifice) are referred to as “CR-associated,” and neurons expressing both intranuclear foci and cytoplasmic *Arc* mRNA expression (i.e. those activated both 5 and 30 min prior to sacrifice) are referred to as “Dual associated.”

Initially, the DS (house light) remained illuminated until the EXP rat pressed the active lever (**Figure 1A**). Upon lever press, the DS extinguished, and the rat received a cocaine infusion followed by a 5-min, non-signaled time out. After the time out, the DS was relit until the rat engaged in another lever press, resulting in the same cocaine infusion/time-out sequence. When a rat self-administered ≥10 infusions within one session, the duration of DS illumination decreased to two min. A lever press during DS illumination resulted in a cocaine infusion/time-out sequence. Failure to respond during DS presentation resulted in a time out. When a rat self-administered ≥10 infusions within one session, the time out was increased to a variable interval, 8-min schedule (VI8; mean  = 8 min, range 6 to 10 min). Training continued until each EXP rat achieved self-administration stability criterion (<15 coefficient of variation in cocaine infusions across three consecutive sessions).

### Conditioned reinforcer

After an active-lever press (**Figure 1B**), EXP rats received a cocaine infusion paired with the complex CR – simultaneous illumination of tricolor LEDs and presentation of a pure tone (2 kHz, 78 dB). The CR remained on for 20 sec. After the CR extinguished, the rat entered a 20-sec, non-signaled time out. After the time out, an active-lever press again resulted in a cocaine infusion/CR/time-out sequence on an FR1 schedule. Once each EXP rat self-administered ≥10 cocaine infusions in a single session, response requirements increased to FR3, then FR5. Daily training continued until stable responding, as defined above, was achieved.

### Extinction

Upon reaching criterion for stable responding during both CR and DS training sessions, EXP rats and controls entered the extinction phase. During extinction, all rats (except CCs) were placed in the chambers for 14 daily, 4.5-hr sessions. Neither the DS nor the CR was given to any rat during extinction. Lever pressing had no consequence.

### Reinstatement test

After the 14th extinction session, all rats remained in the chambers overnight to avoid handling effects on IEG expression (**Figure 1C**). Levers were available, but presses had no consequence. The next day, when the EXP animal pressed the active lever, the CR was presented for 5 sec. For the next 5 min, the CR was presented for two sec contingent on FR1 active-lever pressing. After the 5-min CR-exposure period, there was a 20-min, non-signaled time out. Then, the DS was illuminated for two min, extinguished for one min, re-illuminated for two min, and extinguished. Each cohort (EXP, NC, SC, and DC) received identical exposure to the stimuli contingent on each EXP animal initiating the reinstatement session. Data were statistically analyzed via a two-within (session segment, lever) by one-between (treatment group) mixed-factor ANOVA using JMP10 (SAS Institute Inc.). Planned comparisons between EXP group active- and inactive-lever pressing in the CR and DS segments and EXP group active-lever pressing in the CR and DS segments relative to the extinction and delay periods, as well as relative to control groups during the CR and DS segments were analyzed by Bonferroni-corrected student's t-tests.

### Fluorescent *in-situ* hybridization

After the final DS exposure, all rats were sacrificed via exposure to CO_2_ for one min and then decapitated. Brains were rapidly removed (∼90 sec) and frozen in isopentane (−80°C). Brains were cryosectioned at 12 µm and thaw mounted on Superfrost Plus slides (VWR International). Slides for direct comparison were processed together through all steps, as previously described [32], [33].

### Imaging

**Figure 2:**
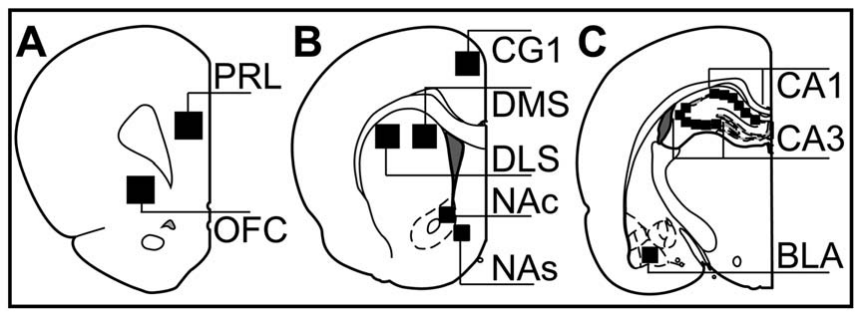
Brain regions analyzed. Coronal diagrams of rat brains show the areas in which gene expression was analyzed, black boxes to scale. (**A**) 0.5 mm^2^ areas in prelimbic (**PRL**) cortex and orbitofrontal cortex (**OFC**). (**B**) 0.5 mm^2^ areas in anterior cingulate cortex (**CG1**), dorsomedial striatum (**DMS**) and dorsolateral striatum (**DLS**), and 0.2 mm^2^ areas in nucleus accumbens core (**NAc**) and shell (**NAs**). (**C**) 0.2 mm^2^ areas in basolateral amygdala (**BLA**) and 0.3 mm^2^ areas in the subregions (**CA1**, **CA3**) of the hippocampus. Diagrams modified and reprinted with permission [53].

Brain sections were visualized using a scanning confocal microscope (Olympus FVX; IX70). Images were captured with a 60X, 1.2-NA oil-immersion objective. A three-by-three montage of 235 µm by 235 µm fields was compiled for dorsomedial (DMS **Figure 2B**) and dorsolateral striatum (DLS **Figure 2B**). A two-by-two montage of 235 µm by 235 µm fields was compiled for nucleus accumbens core (NAc **Figure 2B**), nucleus accumbens shell (NAs **Figure 2B**), and basolateral amygdala (BLA **Figure 2C**). A montage of 6 semi-linear 235 µm by 235 µm fields was compiled for the CA1 and CA3 subregions of dorsal hippocampus (**Figure 2C**). Cell counts were summed across fields and averaged across two sections from each animal for each region. Each field consisted of ten, one-µm thick optical sections, scanned sequentially by 488-nm and 543-nm lasers to resolve Sytox Green (nuclear stain) and Cy3 (*Arc* mRNA) fluorescence, respectively. Sections from dorsal striatum and nucleus accumbens were also scanned at 633-nm to resolve Cy5 (*PPE* mRNA) fluorescence for phenotypic identification of striatal efferent neurons.

### Cellular compartment analysis

An experimenter blinded to treatment condition counted the number of neurons in each field with one or two intranuclear foci of *Arc* mRNA expression, cytoplasmic *Arc* mRNA expression, or both intranuclear and cytoplasmic *Arc* mRNA expression (**Figure 1C**), as previously described [32], [33]. All *Arc* mRNA-positive cells in striatum and nucleus accumbens were also phenotypically identified based on preproenkephalin (*PPE)* mRNA expression. However, analysis revealed no differences in *Arc* mRNA expression in *PPE*-positive and *PPE*-negative cells, and thus *Arc* mRNA-positive cells were not phenotypically differentiated for the final analysis. Data were statistically analyzed via one-way ANOVA for treatment group for each gene-expression profile in each brain region using JMP10 (SAS Institute Inc.).

### Radioactive *in situ* hybridization for Arc and zif268 mRNAs

Brains were cryosectioned at 12 µm and thaw mounted on Superfrost Plus slides (VWR International). Slides for direct comparison were processed together through all steps, as previously described [34].

### Analysis of autoradiograms

Digitized images of 705 µm by 705 µm fields (equivalent to three-by-three catFISH montage) of prelimbic (PRL **Figure 2A**), orbitofrontal (OFC **Figure 2A**), anterior cingulate (CG1 **Figure 2B**), and DMS and DLS (**Figure 2B**) were captured from film autoradiograms. Likewise, digitized images of 470 µm by 470 µm fields (equivalent to two-by-two catFISH montage) of NAc and NAs (**Figure 2B**) and BLA (**Figure 2C**) were captured. Digitized images were also captured from 6 semi-linear 235 µm by 235 µm fields (catFISH equivalent) for CA1 and CA3 (**Figure 2C**) subregions of the dorsal hippocampus. Three brain sections ≥100 µm apart were analyzed bilaterally, yielding 6 analyzed tissue samples for each animal for each region. Digitized Images from film autoradiograms had an 8-bit optical density threshold set such that the lower level was set to the densitometric measurement value of white matter and the upper level remained at 255. After thresholding, brain regions were analyzed using the “integrated density” measure in Image J (http://rsbweb.nih.gov/ij), yielding the product of area sampled and mean gray value for pixels in the field with signal above threshold. These measurements were summed across the 6 samples analyzed in each region for each animal. Values are thus reported as mean ± SEM of integrated density for signal above threshold for each brain region for each treatment group. Data were statistically analyzed via one-way ANOVA for treatment group for each brain region using JMP10 (SAS Institute Inc.). *Post-hoc* analysis of significant ANOVAs was accomplished via the Tukey-Kramer honestly significant difference (HSD) test. Statistical significance was set at p<0.05.

## Results

### Behavior

**Figure 3:**
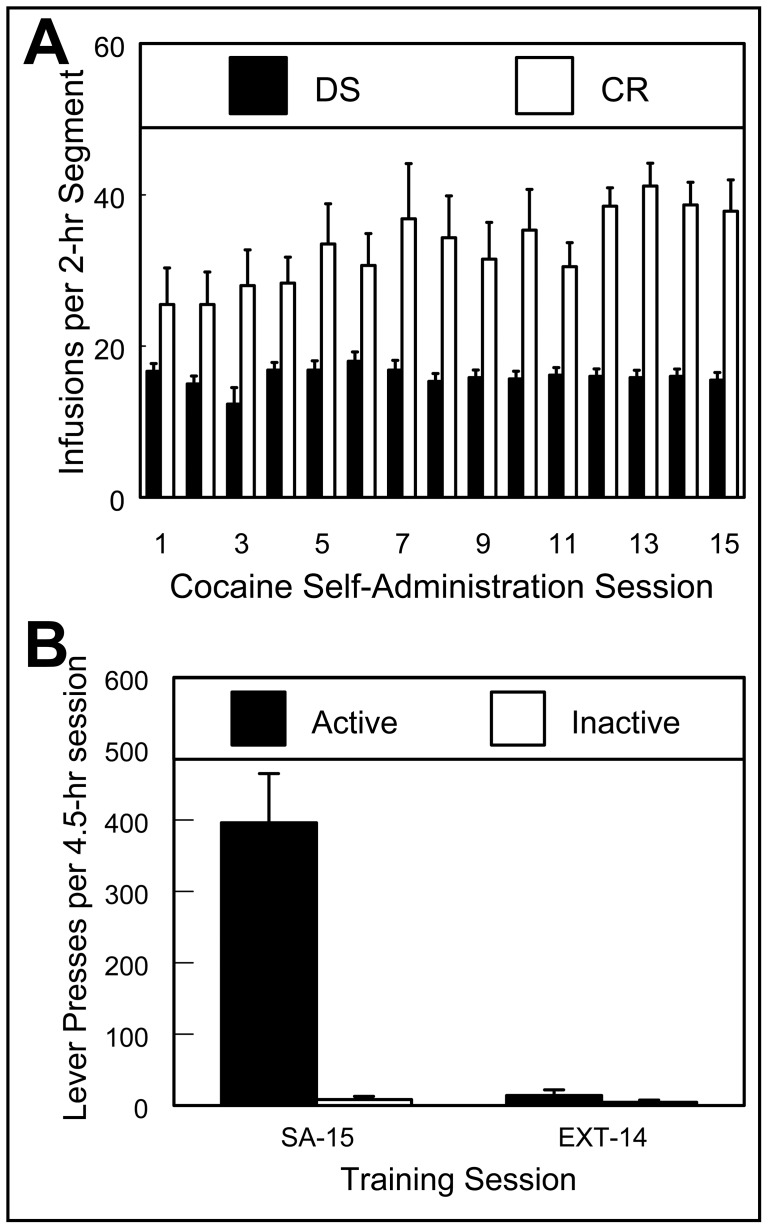
Cocaine self-administration and extinction behavior. Experimental rats earned high and stable levels of self-administered cocaine infusions (0.25 mg cocaine-HCL/50 µL 0.9% saline/2-sec) in both DS- and CR-associated session segments and required approximately 25 sessions for daily infusion rates to stabilize. (**A**) The 15 sessions preceding and culminating in attainment of criterion (3 consecutive sessions with <15 coefficient of variability) are shown. In addition to demonstrating high levels of active-lever pressing during self-administration (**B**; **SA-15**), EXP rats also extinguished active-lever pressing by the last of 14 extinction sessions (**B**; **EXT-14**). Data are means ± SEM for (**A**) numbers of cocaine infusions per two-hour segment and (**B**) lever presses per 4.5-hour session.

As previously reported in a preliminary communication of these results [35], rats acquired stable cocaine self-administration under the dual DS/CR protocols described above (**Figure 1A, 1B**). Of the 15 EXP rats entering training, 10 reached criterion after 24.9±2.6 consecutive daily sessions (mean ± SEM; **Figure 3A** shows average cocaine infusions ± SEM across the last 15 self-administration sessions). Five EXP rats were lost due to blocked catheters. The 10 remaining EXP rats then extinguished lever pressing over 14 extinction sessions (**Figure 3B**).

**Figure 4:**
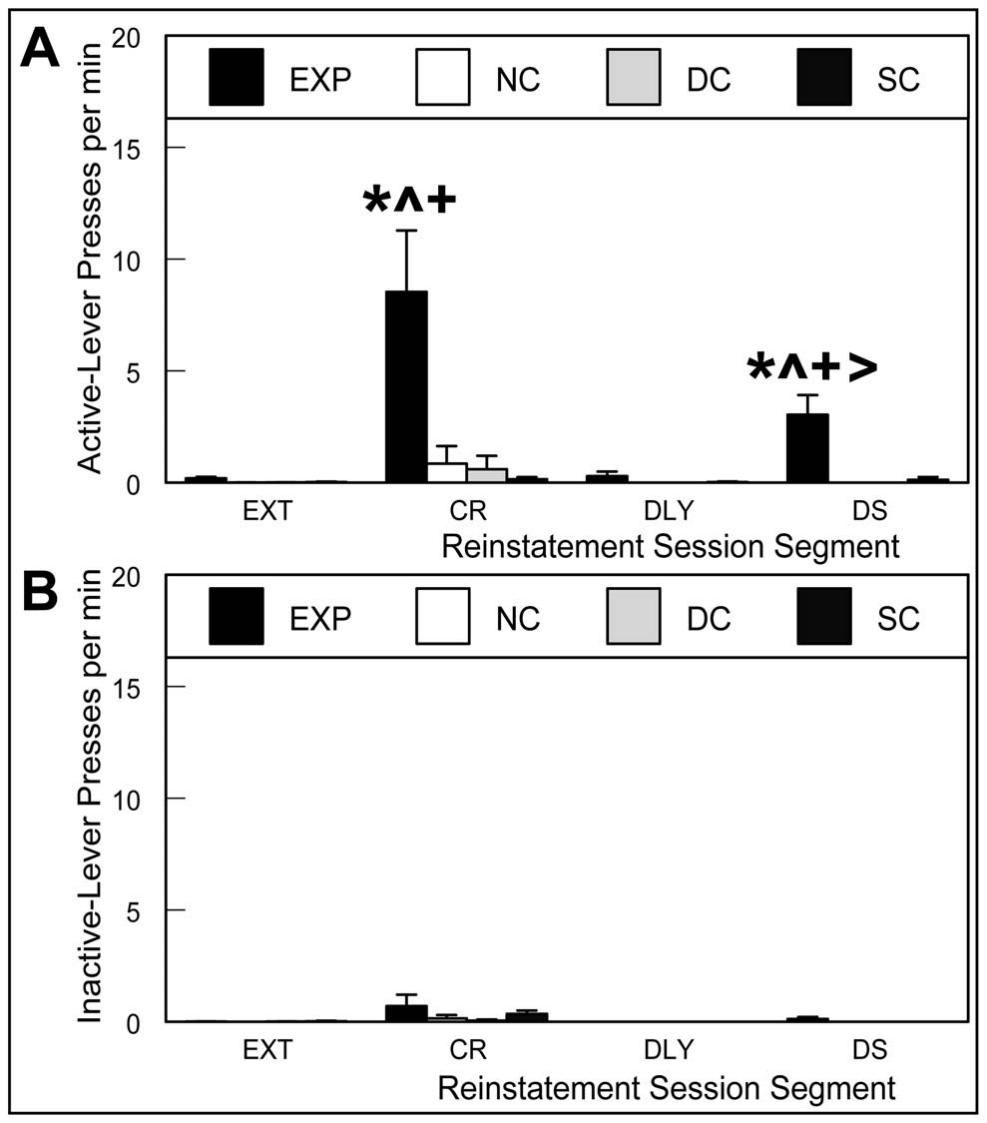
Cue-induced reinstatement of drug-seeking behavior. Rates (per min) of (**A**) active- and (**B**) inactive-lever presses demonstrated during the last of 14 daily extinction sessions (**EXT**), the first 5-min segment of the reinstatement test session in which the conditioned reinforcer (**CR**) was presented contingent on active-lever pressing, the 20-min delay (**DLY**) and during the second 5-min segment in which the discriminative stimulus (**DS**) predictive of cocaine availability was presented twice for two min each, with one min intervening. Experimental rats showed highly significant increases in active-lever pressing in the CR- and DS-associated segments of the reinstatement session compared to inactive-lever pressing, their own previous extinction session, and to all controls within each stimulus exposure segment. Experimental group active-lever pressing was also significantly elevated in the DS segment compared to the 20-min delay. Data are means ± SEM for lever pressing per min for the experimental (**EXP**), novelty control (**NC**), drug control (**DC**), and stimulus control (**SC**) groups.* EXP CR/DS active vs. inactive lever, p<0.01; ** ˆ** EXP CR/DS active vs. EXT, p<0.01; **+** EXP CR/DS active vs. NC, DC, SC, p<0.01; **>** EXP DS active vs. DLY, p<0.01.

Of the 10 EXP rats reaching criterion on the dual training paradigm, 6 reinstated drug-seeking behavior in response to both the DS and CR on the reinstatement test day, with reinstatement being defined as ≥1 lever press per minute during each of the CR and DS segments. EXP rats that did not meet this criterion during both stimulus-exposure segments of the reinstatement test were excluded from the subsequent gene expression analysis. Of the 6 EXP rats reinstating drug-seeking behavior in response to both the DS and CR on test day, four were run with control cohorts and two were from the additional cohort consisting of only EXP animals. Analysis of the reinstatement test session with a two-within (session segment, lever) by one-between (treatment group) mixed-factor ANOVA for lever pressing revealed a significant three-way interaction between treatment group, session segment, and lever (**Figure 4**; F_9,42_ = 3.49, p<0.01). Planned comparisons via Bonferroni-corrected student's t-tests revealed significant increases in active- vs. inactive-lever pressing for the EXP group during both the CR (DF_56_ t_9.0_, p<0.01) and DS (DF_56_ t_3.4_, p<0.01) exposure segments of the reinstatement test session. EXP active-lever pressing was also significantly increased relative to the previous extinction session (CR vs. EXT (DF_84_ t_9.2_, p<0.01); DS vs. EXT (DF_84_ t_3.1_, p<0.01)), as well as relative to the 20-min delay for DS active-lever pressing (DS vs. DLY (DF_84_ t_3.0_, p<0.01). EXP active-lever pressing was further significantly increased when compared to all control groups within reinstatement session segments (CR: EXP vs. NC (DF_111_ t_7.6_, p<0.01), SC (DF_111_ t_8.3_, p<0.01), DC (DF_111_ t_7.9_, p<0.01); DS: EXP vs. NC (DF_111_ t_3.0_, p<0.01), SC (DF_111_ t_2.9_, p<0.01), DC (DF_111_ t_3.0_, p<0.01)). These data demonstrate that EXP rats acquired stable self-administration of cocaine in the context of two distinct sensory-cue associations, extinguished responding to the context of the self-administration chamber over 14 days, and then reinstated drug-seeking behavior in response to temporally discrete presentations of the specific, drug-associated sensory cues.

### CatFISH analysis of *Arc* mRNA expression

The behavioral data presented establish that distinct cocaine-associated CRs and DS support reinstatement of cocaine-seeking behavior when presented in a temporally distinct manner to individual rats. We then asked whether it was possible to use catFISH to assess the transcriptional activation in neural populations associated with this reinstatement. For clarity, neurons determined to be *Arc* mRNA-positive by catFISH analysis are presented as transcriptionally activated at the time of the corresponding segment of the reinstatement session (**Figure 1C**). That is, neurons expressing only intranuclear foci of *Arc* mRNA (i.e. activated 5 min prior to sacrifice) were considered “DS-associated,” neurons expressing only cytoplasmic *Arc* mRNA (i.e. activated 30 min prior to sacrifice) were considered “CR-associated,” and neurons expressing both intranuclear foci and cytoplasmic *Arc* mRNA expression (i.e. activated both 5 and 30 min prior to sacrifice) were considered “dual-associated.” Caged control subjects were never exposed to the reinstatement test stimuli and are not showing transcription related to stimuli exposure, rather these animals demonstrate basal levels of *Arc* mRNA expression. The tissue from one cohort was not available and was not analyzed via catFISH for the DMS, DLS, NAc, and NAs. Tissue from this cohort was analyzed for CA1, CA3, and BLA.

**Figure 5:**
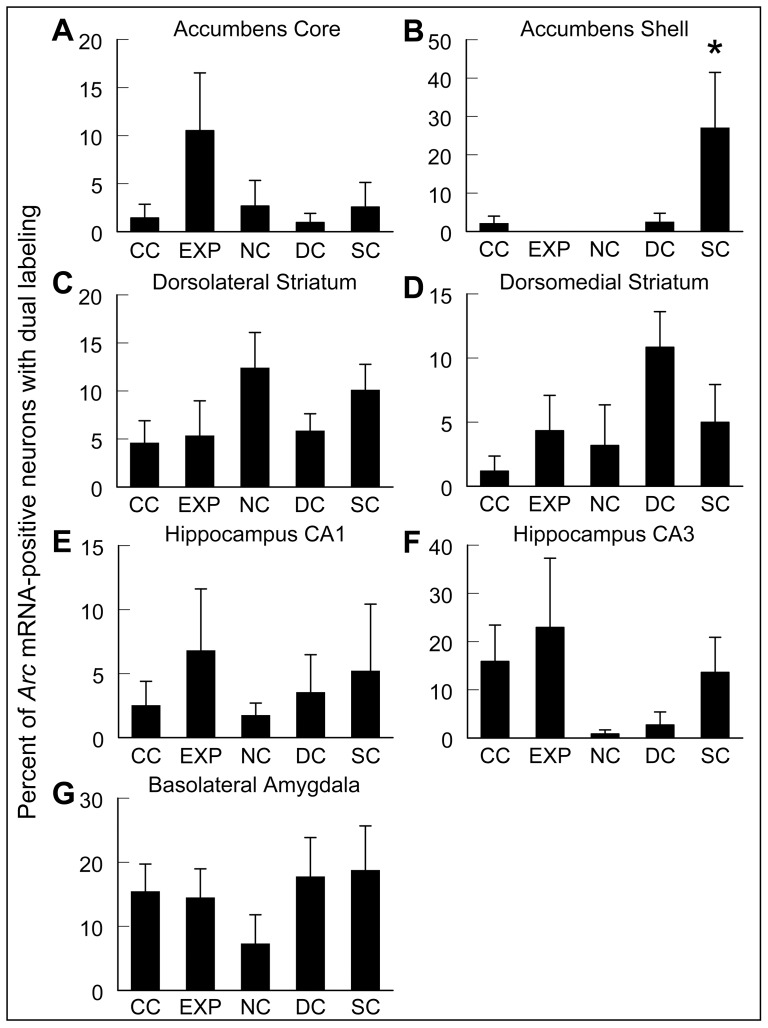
Percentage of *Arc* mRNA-positive neurons showing dual nuclear and cytoplasmic expression (“dual-associated”). Percentage of *Arc mRNA*-positive neurons with dual (*i.e*. both nuclear and cytoplasmic) *Arc* mRNA expression by region analyzed in caged control (**CC**), experimental (**EXP**), novelty control (**NC**), drug control (**DC**), and stimulus control (**SC**) rats (see Methods for full description of different groups). Data are mean percentage ± SEM (n = 4–6) of *Arc* mRNA-positive neurons showing dual-associated expression. * Significantly different from all other groups, p<0.05.

One-way ANOVAs for each brain region analyzed revealed no significant effects of treatment group on the number of *Arc* mRNA-positive neurons activated at the time of the DS session (DS-associated), the CR session (CR-associated), or both segments of the reinstatement test session (Dual-associated) in the DMS, DLS, NAc or NAs, CA1 or CA3 subregions of the hippocampus, or the BLA (**Table 1**). Furthermore, as shown in **Figure 5**, the proportion of neurons with dual *Arc* mRNA expression (i.e. those with nuclear foci and cytoplasmic *Arc* mRNA expression) was very low across all brain regions examined in the EXP, as well as all other control groups. One-way ANOVAs failed to show significant effects of treatment group on the proportion of neurons with dual *Arc* mRNA expression for all brain regions except the NAs. Analysis of the NAs revealed a main effect of treatment group (**Figure 5B**; F_4,18_ = 4.3, p<0.05). *Post-hoc* analysis by Tukey-Kramer HSD (p<0.05) revealed that the percentage of *Arc* mRNA-positive cells with dual expression in the SC group was significantly greater than in the CC, EXP, and NC groups. Notably, however, the robust reinstatement of cocaine-seeking behavior elicited by temporally discrete exposure to CR and DS cues in the EXP animals was not associated with significant increases in the numbers of *Arc* mRNA-positive neurons in any of these brain regions, nor a change in the proportion of neurons (i.e. neural ensembles) showing dual (nuclear plus cytoplasmic) *Arc* mRNA expression.

**Table 1:** Catfish results.

			*Arc* mRNA-positive neurons per 0.5 mm^2^					
			CR-associated		DS-associated		Dual-associated	
Region	Sub-region	Group	Mean	SEM	Mean	SEM	Mean	SEM
Striatum	Dorsomedial	CC	0.9	1.4	6.8	4.2	0.1	0.7
		**EXP**	**3.9**	**1.4**	**17.8**	**4.2**	**1.6**	**0.7**
		NC	3.8	1.8	14.2	5.4	1.0	1.0
		SC	5.3	1.8	7.7	5.4	1.0	1.0
		DC	6.2	1.8	8.7	5.4	2.0	1.0
	Dorsolateral	CC	19.3	11.0	10.1	3.8	2.7	2.8
		**EXP**	**18.4**	**11.0**	**18.8**	**3.8**	**4.4**	**2.8**
		NC	31.2	14.2	16.5	4.9	8.7	3.7
		SC	16.8	14.2	15.7	4.9	3.8	3.7
		DC	35.7	14.2	13.7	4.9	3.5	3.7
Nucleus Accumbens	Core	CC	0.2	1.3	10.6	4.5	0.2	0.6
		**EXP**	**2.0**	**1.3**	**14.9**	**4.5**	**1.8**	**0.6**
		NC	3.8	1.7	11.6	5.8	0.8	0.7
		SC	2.6	1.7	11.6	5.8	0.4	0.7
		DC	6.0	1.7	18.4	5.8	0.4	0.7
	Shell	CC	0.9	1.0	5.2	3.0	0.2	0.4
		**EXP**	**1.6**	**1.0**	**7.7**	**3.0**	**0.0**	**0.4**
		NC	2.6	1.3	6.4	3.8	0.0	0.6
		SC	2.3	1.3	4.9	3.8	1.9	0.6
		DC	3.8	1.3	9.0	3.8	0.4	0.6
Hippocampus	CA1	CC	4.9	6.0	14.8	5.2	1.4	2.5
		**EXP**	**9.5**	**6.0**	**19.6**	**5.2**	**5.1**	**2.5**
		NC	3.9	7.4	5.5	6.4	0.3	3.1
		SC	6.9	7.4	3.4	6.4	2.3	3.1
		DC	8.4	7.4	10.9	6.4	1.5	3.1
	CA3	CC	4.9	5.6	9.8	3.5	2.5	1.8
		**EXP**	**9.3**	**5.6**	**12.0**	**3.5**	**2.1**	**1.8**
		NC	10.3	6.9	4.3	4.2	0.6	2.2
		SC	10.3	6.9	12.0	4.2	6.8	2.2
		DC	5.6	6.9	4.1	4.2	0.8	2.2
Amygdala	Basolateral	CC	14.5	5.7	14.4	6.5	8.6	3.8
		**EXP**	**12.8**	**5.7**	**14.8**	**6.5**	**7.3**	**3.8**
		NC	6.1	7.0	6.5	8.0	2.1	4.7
		SC	6.8	7.0	10.9	8.0	7.0	4.7
		DC	6.1	7.0	6.0	8.0	2.3	4.7

### Autoradiographic *in situ* hybridization

Given the lack of increase in the numbers of *Arc* mRNA-positive neurons in EXP animals relative to controls in any subcellular compartment, we became concerned that the amplification steps in the catFISH assay might be masking differences in the relative amount of *Arc* mRNA expression per cell across treatment groups, as several studies using radioactive *in situ* hybridization to examine gene expression in the context of cue-induced reinstatement of cocaine-seeking behavior have reported increases in *Arc* and *zif268* mRNA expression [19], [36], [37]. Therefore, to address whether nonlinearities introduced by the amplification steps in the catFISH approach were obscuring differences across treatment groups, we analyzed additional sets of tissue using standard radioactive *in situ* hybridization histochemistry in which the signal detected is directly related to the amount of probe bound.

### *Arc* mRNA expression

**Figure 6:**
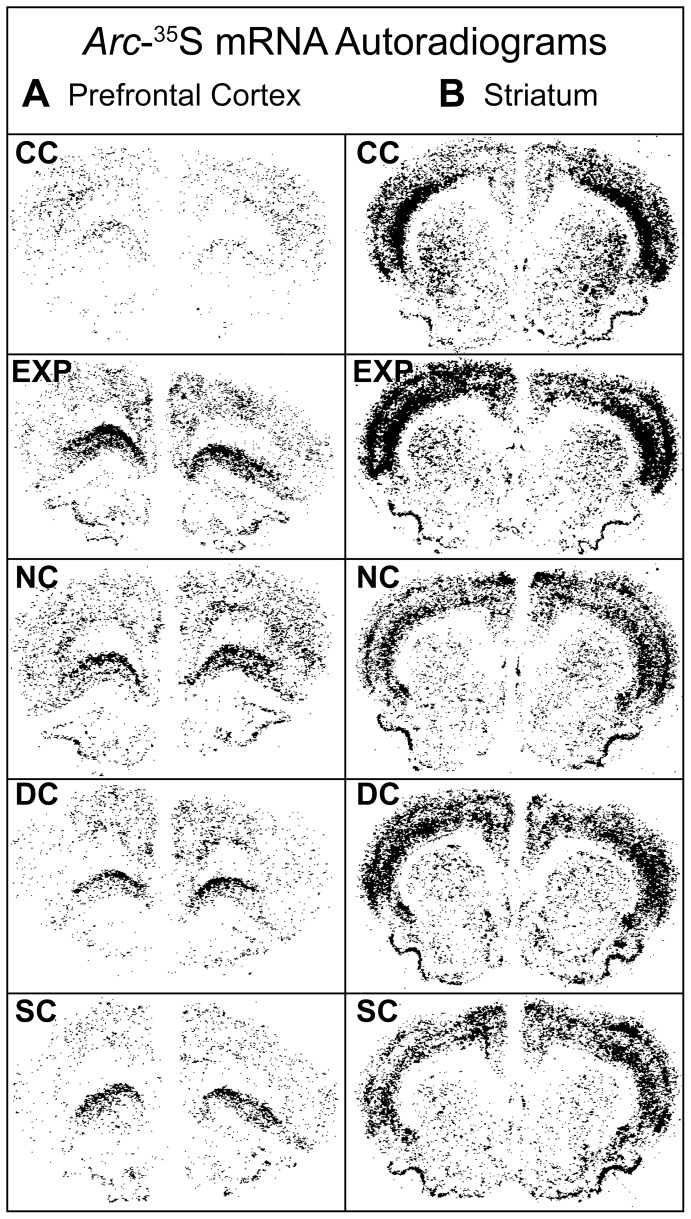
Representative images from radioactive *in situ* hybridization film autoradiograms of *Arc-^35^S* mRNA expression. Images of film autoradiograms from a single cohort of animals showing *Arc-^35^S* mRNA expression in black for caged control (**CC**), experimental (**EXP**), drug control (**DC**), stimulus control (**SC**), and novelty control (**NC**) animals in various subregions of the (**A**) prefrontal cortex and (**B**) striatum.

**Figure 7:**
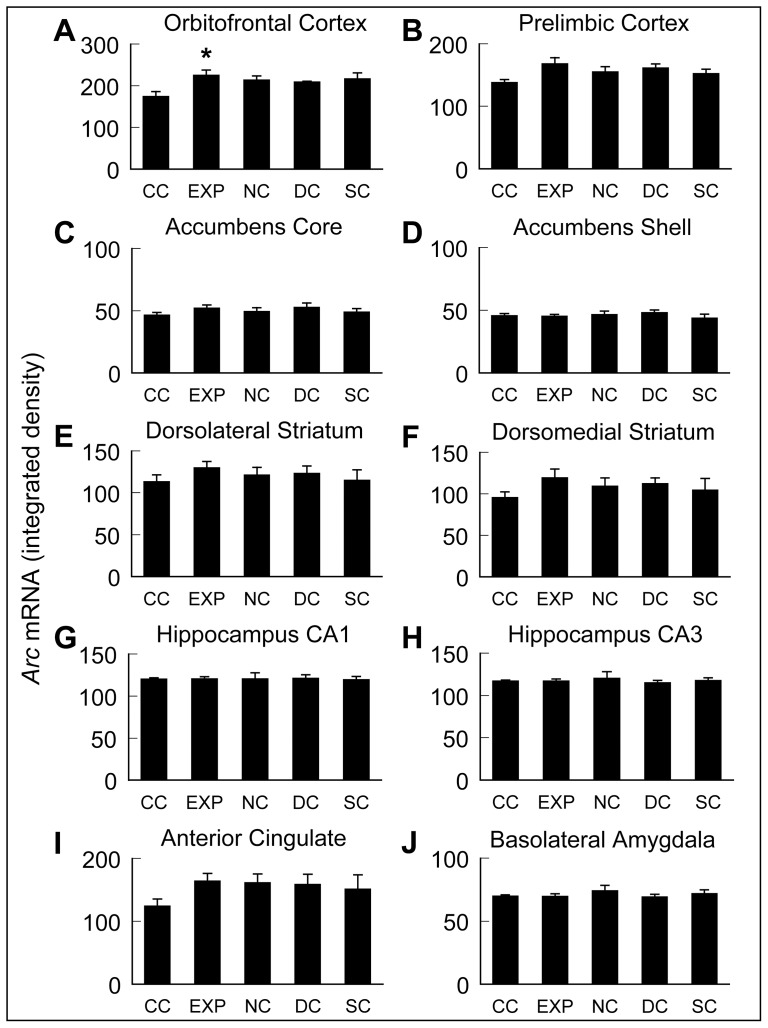
Densitometric analysis of *Arc* mRNA expression. *Arc* mRNA expression (integrated density) determined from analysis of film autoradiograms. Data are means ± SEM (n = 4–6/group). * Significantly different from CC, p<0.05.

A one-way ANOVA revealed no significant effect of treatment group on the density of *Arc* mRNA expression in CG1, NAc, NAs, DLS, DMS (**Figure 6B** **,** **7**), or CA1, CA3, or BLA (**Figure 7**). A one-way ANOVA for treatment group on the density of Arc mRNA expression in PRL (**Figure 7B**) showed a strong trend (F_4,23_ = 2.4, p = 0.08) towards significance.

A one-way ANOVA did reveal a significant main effect of treatment group on the density of *Arc* mRNA expression in OFC (F_4,23_ = 3.3, p<0.05; **Figure 6A** **,** **7A**). *Post-hoc* analysis via Tukey-Kramer HSD (p<0.05) confirmed increased density of *Arc* mRNA in experimental animals relative to caged controls, but this increase was not significantly different from other operant chamber exposed groups (NC, DC, SC).

### *Zif268* mRNA expression

**Figure 8:**
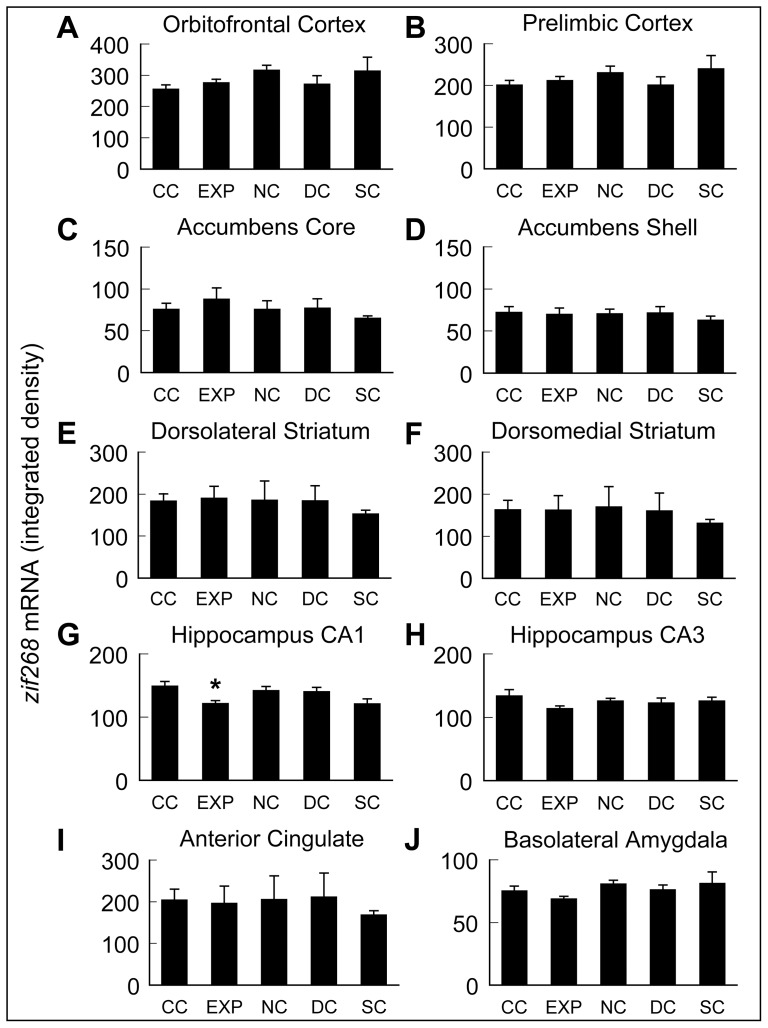
Densitometric analysis of *zif268* mRNA expression. *zif268* mRNA expression (integrated density) determined from analysis of film autoradiograms. Data are means ± SEM (n = 4–6/group). * Significantly different from CC, p<0.05.

A one-way ANOVA revealed no significant effect of treatment group on z*if268* mRNA expression in OFC, PRL, CG1, NAc, NAs, DLS, DMS, or CA3 (**Figure 8**).

One-way ANOVA did reveal a significant main effect of treatment group on *zif268* mRNA expression in CA1 (F_4,23_ = 3.7, p<0.05; **Figure 8G**). *Post-hoc* analysis via Tukey-Kramer HSD testing (p<0.05) confirmed a significant decrease in the expression of *zif268* mRNA in experimental animals in the opposite direction of predicted results.

## Discussion

The first goal of these experiments was to determine the feasibility of training rats to self-administer cocaine in a segregated DS/CR model. The results show that rats can learn to stably self-administer cocaine in such a dual paradigm (**Figure 3**), with 40% of rats successfully reinstating lever pressing to both cues on the test-day. The second goal was to identify neural ensembles associated with cue-induced reinstatement of cocaine seeking using catFISH, which affords high sensitivity, temporal fidelity, and cellular resolution [23]. Unexpectedly, we failed to find increases in the numbers of neurons expressing *Arc* mRNA or changes in the proportions of neurons expressing *Arc* mRNA in response to dual DS/CR cue-induced reinstatement of cocaine seeking in experimental rats. Subsequent autoradiographic analysis of the expression of *Arc* and *zif268* mRNAs also failed to reveal increases in the expression of these genes associated with the reinstatement of drug-seeking behavior induced by exposure to drug-associated cues in EXP animals relative to controls. These data demonstrate that drug-seeking behavior evoked by brief exposure to discrete sensory cues is not associated with detectable increases in the transcriptional activation of *Arc* or *zif268* mRNAs in multiple brain regions, suggesting that neither *Arc* nor *zif268* mRNA expression, in the regions analyzed, is necessary for discrete cue-induced reinstatement of drug-seeking behavior and that brief exposure to reinstatement may not be sufficient to drive such expression.

Rats learned and reinstated cocaine-seeking behavior in response to two distinct types of stimuli, however the proportion of animals completing training and reinstating to both the CR and the DS was low. Two factors likely contribute to this outcome. First, the number of days required for rats to reach criterion was relatively high. Rats in the present study completing dual DS/CR training required 24.9±2.6 sessions to reach criterion. Other labs utilize 10 consecutive training sessions with ≥10 infusions per session [13], [37]–[40], >10 infusions/day for three consecutive days [17], <10% variability in responding across three sessions [16], or <20% variability in cocaine intake across 5 sessions [41] to establish self-administration behavior. Consequently, one-third of rats entering training failed to reach criterion due to obstruction of the catheter lines after comparatively long periods of training.

The second factor likely contributing to high attrition in the dual DS/CR paradigm is general task difficulty. Two rats reinstated to only one stimulus, and their performance during training was better for the corresponding stimulus. Although both DS and CRs likely contribute to reinstatement and maintenance of drug-seeking in addicted subjects [42], high attrition may limit the use of an animal model to systematically examine the specific roles of stimulus type within an individual. However, despite this difficulty, these data demonstrate the ability to train rats in this manner.

As noted above, the second goal of the present study was to determine whether catFISH could be used to map neuronal ensembles activated by reinstatement of cocaine-seeking behavior by either or both of the cocaine-associated cues (DS and/or CR). The lack of significant increases in the numbers of cells with expression or in the general levels of *Arc* and *zif268* mRNA expression across multiple brain regions in the EXP group relative to other control groups was quite surprising, especially in light of reports of significant increases in the expression of these genes in striatum [37] and prefrontal cortex [19], [36] in rodent models of cue-induced reinstatement of cocaine-seeking behavior and other data heavily implicating *Arc* and *zif268* mRNA expression in learning, memory, and reconsolidation of previously acquired associations [32], [43]–[45].

There are several possible explanations for the lack of specific *Arc* and *zif268* mRNA transcriptional activation in EXP animals in the present work. This reinstatement model is different from those used in other studies showing reinstatement-induced increases in gene expression in that extreme care was taken in the present work to isolate the specific, discrete sensory cues most proximally eliciting reinstatement of cocaine-seeking behavior and to present them for necessarily brief periods of time to capitalize on the strength of the catFISH technique. Thus, the two major differences across the studies reporting increases in IEG expression [19], [36], [37] and the present study are the global salience of the cocaine-associated cues and context and the amount of time the animals were exposed to the cues during the reinstatement test. Hearing et****al. exposed rats to a full hour of reinstatement testing [36], [37], and Zavala and colleagues exposed rats to contingent CR presentation for 30 min [19]. Furthermore, in the work of Hearing et****al. [36], [37], rats were abstinent from the self-administration chambers for two weeks prior to reinstatement testing. These rats were not extinguished to the predictive value of the cocaine-associated context. Thus, all of the stimuli in the operant chamber, especially the chamber itself, were highly salient to the rats on test day. Furthermore, the test day was the first experience of a change in response-contingency for these subjects in that environmental context. In contrast, in the present study animals were exposed to the cues for only 4.5 min and were extinguished to all operant chamber cues other than the drug-associated DS and CR. Importantly, discrete cues presented for short periods were sufficient to elicit rates of reinstatement (**Figure 4**) of cocaine-seeking behavior in EXP animals greater than those reported in the extant literature for the first 5 min of longer reinstatement tests [22], [46], [47]. Thus, an absence of sufficient cue-induced reinstatement behavior does not explain the lack of gene expression in this study.

Exposure to drug-associated cues for a longer period of time may be necessary to induce gene expression in these types of models not because such expression is required for the activation of a learned association, but rather because the gene expression may in fact reflect new extinction learning, or neural plasticity related to alteration of the encoded associations in the context of longer sessions in which cues are no longer paired with drug delivery. In this regard, the data of Hearing and colleagues [48] are informative. In that study infusion of *Arc* mRNA anti-sense oligonucleotides into the striatum failed to alter context-induced reinstatement of cocaine-seeking behavior. However, the following day, rats so treated showed greater reinstatement behavior, suggesting that the *Arc* mRNA anti-sense had impaired consolidation of the extinction learning occurring on the first day of reinstatement testing. Although IEG expression has often been used to map activated neural circuitry, given the role of IEGs in learning, and its consolidation and reconsolidation, careful consideration needs to be given when interpreting the meaning of changes in IEG expression induced during reinstatement of drug-seeking behavior when the contingencies between drug-seeking behavior and drug delivery change on the test day. The new learning occurring under such altered contingencies clearly becomes a significant confound in interpreting the meaning of the associated IEG expression. The use of second-order schedules of reinforcement therefore may be more useful for mapping, *via* IEG expression, brain circuitry activated during drug seeking, as such schedules engender significant drug-seeking behavior in the absence of primary reinforcement and without altering existing contingencies.

Generally, there were limited differences in *Arc* or *zif268 mRNA* expression specific to EXP animals in the present study. There was a significant increase in the level of *Arc* mRNA expression analyzed by radioactive *in situ* hybridization in the OFC of EXP animals compared to the CC group, and a trend towards significance in PRL. The OFC has been shown to be important for drug context-induced reinstatement of drug seeking [49]. Furthermore, the OFC is purported to be a critical component of an inhibitory response control system involving the dorsal striatum and the subthalamic nucleus [50]. In light of this information one might speculate that the increased expression of *Arc* mRNA in the OFC of EXP animals observed herein may be evidence that new learning is taking place in these structures, as the reinstatement test is, in effect, an extinction session. However, the fact that *Arc* mRNA expression in the EXP group was not significantly different from that in the other control groups exposed to the discrete sensory cues on the test day (NC, SC, DC) suggests that increases in *Arc* mRNA expression in OFC may simply reflect general behavioral activation induced by exposure to environmental stimuli, independent of any association those cues have with prior drug taking. A recent analysis of *Arc* mRNA expression in mice undergoing CR-induced reinstatement of cocaine-seeking behavior found increased levels of *Arc* mRNA only in prelimbic cortex [51], consistent with our findings. The prelimbic cortex and adjacent infralimbic cortex have been shown to be critically important in the encoding and expression of behavioral extinction both in the context of reinstatement of cocaine seeking as well as fear conditioning, see [52] for review. Rather surprisingly, the CA1 region of the hippocampus showed a significant overall difference between groups in *zif268 mRNA* expression, with *post-hoc* analysis showing *zif268* mRNA expression to be significantly lower in the EXP group relative to the caged controls. This decrease was unexpected. Our data are thus the first to suggest suppression of *zif268* mRNA expression in a reinstatement model, although the functional significance of this decrease remains to be determined.

## Conclusions

In conclusion, the experiments described herein show that individual rats can be trained with discrete associative mechanisms and then reinstate drug-seeking behavior in response to each type of drug-associated cue. The data further suggest that such reinstatement of drug-seeking behavior is not necessarily associated with elevated transcriptional activation of the IEGs *Arc* and *zif268* across multiple brain regions heavily implicated in cue-induced reinstatement of drug-seeking behavior. These data therefore emphasize the need to carefully consider not only the role of drug-seeking behavior, but also that of new learning that is likely occurring in drug-reinstatement paradigms, when interpreting changes in IEG expression associated with reinstatement of drug-seeking behavior.
